# *Aphelenchoides besseyi* Ab-FAR-1 Interacts with *Arabidopsis thaliana* AtADF3 to Interfere with Actin Cytoskeleton, and Promotes Nematode Parasitism and Pathogenicity

**DOI:** 10.3390/ijms232012280

**Published:** 2022-10-14

**Authors:** Shanwen Ding, Xi Cheng, Dongwei Wang, Chun Chen, Sihua Yang, Jiafeng Wang, Chunling Xu, Hui Xie

**Affiliations:** 1 Laboratory of Plant Nematology and Research Center of Nematodes of Plant Quarantine, Department of Plant Pathology, College of Plant Protection, South China Agricultural University, Guangzhou 510642, China; 2 Guangdong Provincial Key Laboratory of High Technology for Plant Protection, Institute of Plant Protection, Guangdong Academy of Agricultural Sciences, Guangzhou 510640, China; 3 Key Laboratory of Biopesticide and Chemical Biology, Ministry of Education, College of Plant Protection, Fujian Agriculture and Forestry University, Fuzhou 350002, China; 4 Hunan Plant Protection Institute, Hunan Academy of Agricultural Science, Changsha 410125, China

**Keywords:** plant parasitic nematodes, fatty acid and retinol binding protein, parasitism and pathogenicity, actin cytoskeleton, plant immunity

## Abstract

Fatty acid and retinol binding proteins (FAR) are unique proteins found in nematodes and are considered potential targets for controlling these parasites. However, their functions in nematode parasitism and pathogenicity and interaction with hosts are still unclear. In this study, we investigated the specific roles of rice white tip nematodes (RWTNs), *Aphelenchoides besseyi*, and a protein, Ab-FAR-1, to elucidate the parasitic and pathogenic processes of nematodes. The results showed that the expression level of *Ab-far-1* was significantly up-regulated after *A. besseyi* infection of the plant. The immunofluorescence and subcellular localisation showed that Ab-FAR-1 was secreted into plant tissues mainly through the body wall of nematodes and might act in the nucleus and cytoplasm of plant cells. The pathogenicity of RWTNs was enhanced in *Arabidopsis thaliana* overexpressing Ab-FAR-1 and inhibited in *Ab-far-1* RNAi A. thaliana. Yeast two-hybrid, Co-IP, BiFC, and nematode inoculation experiments showed that Ab-FAR-1 could interact with the *A. thaliana* actin-depolymerizing factor protein AtADF3, and the *A. thaliana adf3* mutant was more susceptible to nematodes. An in vitro actin filament depolymerisation assay demonstrated that Ab-FAR-1 could inhibit AtADF3-mediated depolymerisation of actin filaments, and the turnover process of cellular actin filaments was also affected in *A. thaliana* overexpressing Ab-FAR-1. In addition, flg22-mediated host defence responses were suppressed in *A. thaliana* overexpressing Ab-FAR-1 and adf3 mutants. Therefore, this study confirmed that RWTNs can affect the turnover of actin filament remodelling mediated by AtADF3 through Ab-FAR-1 secretion and thus inhibit plant PAMP-triggered immunity (PTI), promoting the parasitism and pathogenicity of nematodes.

## 1. Introduction

The economic losses caused by plant-parasitic nematodes are estimated at USD 173 billion annually worldwide [1]. The rice white tip nematode (RWTN) *Aphelenchoides besseyi* is a parasitic nematode that is widely present in crops, and more than 200 plant species can act as hosts for these nematodes. RWTN is widely distributed in almost all rice-growing areas in the world, causing losses of up to 50% in flooded rice [2]. It is difficult to implement a good means for thorough control of RWTN once it outbreaks. It is one of the top ten plant-parasitic nematodes and has been listed as a quarantined plant pest in many countries [3,4]. Finding more effective and safe control measures is one of the main directions for research focusing on RWTNs and other nematodes.

Unlike animals, plants lack specialised immune cells and rely exclusively on innate immunity to defend themselves against microbial invaders. The plant immune system operates on a sophisticated two-tier system, PAMP-triggered immunity (PTI) and effector-triggered immunity (ETI) [5]. As a dynamically changing three-dimensional network structure in eukaryotic cells, the actin cytoskeleton participates in the basic life activities of cells and facilitates a dynamic reorganisation process under the regulation of various actin-binding proteins (ABPs). In turn, the ABPs are involved in the perception and response of cells to external biotic and abiotic stimuli and balance and resist the threats of external stimuli to cell life activities [6]. In recent years, many studies have shown that the plant actin cytoskeleton is involved in the immune processes of plant PTI and ETI [6,7,8] and plays an important role in plant sensing and pathogen resistance [9]. In the plant–pathogen co-evolution process, pathogens use various strategies to avoid the plant immune response and maintain their virulence. At the same time, plants have evolved new defence strategies to resist the infection of pathogens. This “Zig-Zag” model is currently one of the many focus areas in plant–pathogen interaction research, and the discovery and analysis of its mechanisms will help us to better understand and control plant diseases [5]. 

Plant nematodes can secrete effectors to plant cells through nematode organs, such as the stylet and body wall. This affects the growth and metabolism of plants, dynamic balance of the cytoskeleton in plant cells, and defence responses of the plant. Consequently, these changes create a suitable environment for nematodes to complete their growth and development [10]. Therefore, studying the functions of these effectors and their interactions with hosts will help us to understand the nematode pathogenic mechanism and will allow for the selection of appropriate targets to control plant nematodes. Most studies have focused on the effectors expressed in the oesophageal glands of nematodes or the effectors secreted by their stylet [11]. These effectors are considered to be the key factors in the nematode parasitic process. For example, *Globodera rostochiensis* SPRYSECE-19 and GrUBCEP12, *Meloidogyne incognita* Mi-CRT, *Meloidogyne javanica* MjTTL5, *Meloidogyne graminicola* MgGPP, and *M. graminicola* MgMO237 have been shown to inhibit plant defence responses in different ways [12,13,14,15,16,17,18]. The effector MiPFN3 of *M. incognita* disrupts the actin cytoskeleton of plants to promote parasitism [19]. In addition, some studies have demonstrated that the effectors secreted by nematode body walls are crucial in the infection and pathogenicity of nematodes [20,21]. For example, the peroxidase secreted by the hypodermis of *G. rostochiensis* and *M. incognita* can promote the development of nematodes by scavenging reactive oxygen species (ROS) in the host [22,23]. Furthermore, the MiMIFs of *M. incognita* secreted by the hypodermis can interact with *Arabidopsis thaliana* annexin, suppressing plant immunity and promoting nematode parasitism (Zhao et al., 2019). Fatty acid and retinol-binding proteins (FAR) are among the most widely studied proteins secreted by the body wall of nematodes, and have been widely reported in free-living nematodes, animal-parasitic nematodes, and plant-parasitic nematodes [24,25,26,27,28,29,30,31,32,33,34,35,36,37]. At present, the structures and functions of FAR are mainly explained as follows: (1) FAR are unique to nematodes and do not exist in other biological groups [38]; (2) their function in obtaining fatty acids and retinols from hosts and the environment is necessary for nematode lipid biosynthesis and assembly of macromolecular structures to complete the growth and development of nematodes [31,32]; (3) most of them are located on the nematode body wall, which is the host–parasite interface, and, therefore, they play important roles in promoting nematode parasitism and regulating host defences [30]; and (4) there is evidence that FAR can influence the fecundity and pathogenicity of nematodes [32,33,39]. In previous studies, we found eight FAR proteins with significantly different mRNA localisations and diverse functions in RWTNs [32,33,39]. The expression level of *Ab-far-1* was significantly higher than that of other FAR genes in RWTNs. In vitro RNAi and fungus-mediated RNAi assays showed that silencing of *Ab-far-1* affected the reproduction and pathogenicity of the RWTNs. However, it is still unknown how it affects plant development and defence responses; therefore, it is necessary to study the mechanism of Ab-FAR-1 in the host plant.

In this study, the specific role of Ab-FAR-1 in the interaction between RWTNs and their host *A. thaliana* was further explored. The mechanism of action of Ab-FAR-1 has been revealed through some biological experiments, such as immunofluorescence localization and yeast two-hybrid, etc. These findings will provide new insights into plant–nematode interaction and reveal the target of Ab-FAR-1 in host plants and new mechanisms for inhibiting plant defences.

## 2. Results

### 2.1. Ab-FAR-1 Plays an Important Role in the Process of Nematode Infection 

**Figure 1:**
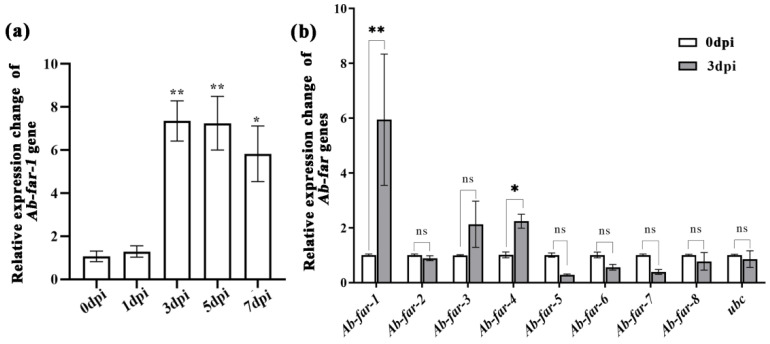
Expression changes of *Ab-far* genes in the process of nematode infection. (**a**) The expression changes of *Ab-far-1* gene during RWTN infection. (**b**) The expression changes of *Ab-far* genes (Ab-far-1 to Ab-far-8) 3 days post-infection (dpi). To determine the silence efficiency of gene, the gene expression from 0 dpi was normalized as 1, and fold change was calculated using the 2^-ΔΔCt^ method to determine a change in expression of the genes of interest. Values are presented as the means ± standard error (SE), *n* = 9. Asterisks denote significant differences (* *p* < 0.05, ** p < 0.01; *t*-test) from the 0 dpi treatment.

*Ab-far-1* expression at 0 to 7 dpi was detected by RT-qPCR in *A. thaliana* (Figure 1a). The expression level of *Ab-far-1* rapidly increased within 1–3 dpi, remained stable from 3–5 dpi, and decreased slightly after 5–7 dpi. The expression levels at 3–7 dpi were significantly higher (*p* < 0.05) than those at 0 dpi. This indicates that *Ab-far-1* might be involved in nematode infection of plants. In addition, the expression changes of eight *Ab-far* genes in RWTNs were detected at 3 dpi (Figure 1b). Only *Ab-far-1* and *Ab-far-4* were significantly up-regulated, and *Ab-far-1* was the most significantly up-regulated of the eight *Ab-far* genes monitored. There were no significant differences between the other six *Ab-far* genes and the control *ubc* gene before and after RWTN infection. Therefore, the *Ab-far-1* gene may play an important role in nematode infection in plants.

### 2.2. Immunological Localisation of Ab-FAR-1^Δsp^

**Figure 2:**
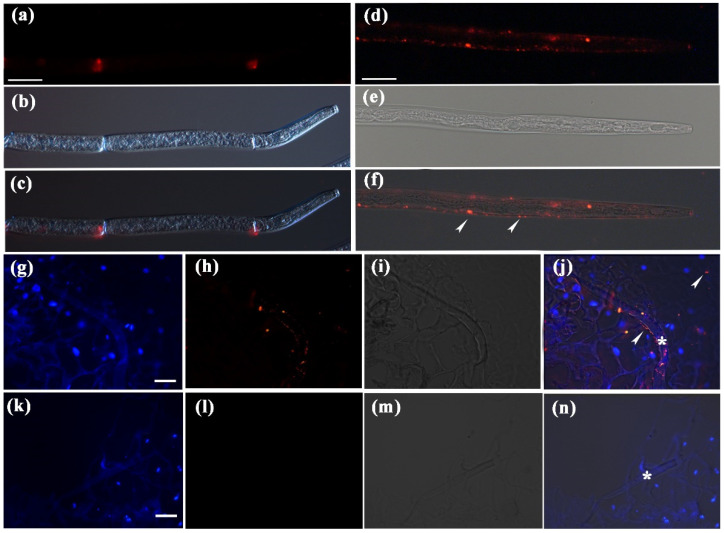
Localisation of Ab-FAR-1 protein in nematode and in planta. (**a**–**c**) No fluorescence signal was found when Ab-FAR-1 antibody was replaced by antiserum before immunization; Ab-FAR-1 protein secreted was located in the body wall of nematodes (**d**–**f**) and host cells (**g**–**j**) (white arrows). Micrographs (**a**,**d**,**h**,**l**) were observations of Alexa Fluor 594-conjugated secondary antibody. Micrographs (**g**,**k**) shows 4, 6-diamidino-2-phenylindole (DAPI)-stained nuclei. Micrographs (**b**,**e**,**i**,**m**) are images of differential interference contrast. Micrographs (**c**,**f**,**j**,**n**) were superpositions of images. The white arrows and asterisks indicate the location of the fluorescence signal and nematodes. Bar = 25 μm.

A previous study showed that *Ab-far-1* mRNA was located in the hypodermis and other part ovaries of nematodes by in situ hybridisation [26]. Signal peptide-carrying protein was transported into the endoplasmic reticulum and Golgi for processing and modification, and it would be cut off during the process of being secreted out of the cell [40]. To further determine the tissue localisation of the Ab-FAR-1 protein and the secretion process after nematode infection, we performed immunofluorescence localisation analysis of Ab-FAR-1 without a signal peptide (Ab-FAR-1^Δsp^) on RWTN bodies and plant sections from *A. thaliana* infected with RWTNs at 3 dpi. First, the 32.14 kDa rAb-FAR-1^Δsp^ protein containing the trxA tag was obtained by prokaryotic expression. We produced polyclonal antibodies against rAb-FAR-1^Δsp^ by immunising rabbits, and the antibody titres were all greater than 512 K (Figure S1a). Western blotting showed that the anti-rAb-FAR-1B polyclonal antibody specifically detected rAb-FAR-1^Δsp^ at the expected size of ~32 kDa and rAb-FAR-1^Δsp^ in nematode proteins at the expected size of ~19 kDa (Figure S1b). No signal was detected in the total proteins from healthy leaves. The size of the band was consistent with the predicted size, indicating that anti-rAb-FAR-1B could specifically recognise Ab-FAR-1^Δsp^. Immunolocalization was performed on the nematode bodies and sections of *A. thaliana* infected with RWTNs. Strong red fluorescent signals of Ab-FAR-1^Δsp^ were observed in the body walls of nematodes (Figure 2f,j) and plant tissue sections (Figure 2j) treated with anti-rAb-FAR-1B serum, but not in the control nematodes (Figure 2c) and plant tissue sections (Figure 2n) treated with pre-immune serum. These results suggest that Ab-FAR-1 is secreted by the body walls of RWTNs and is likely targeted to host tissues. Therefore, it may play an important role in the parasitic processes of RWTNs.

### 2.3. Ab-FAR-1^Δsp^ Localized in Plant Cytoplasm and Nucleus

**Figure 3:**
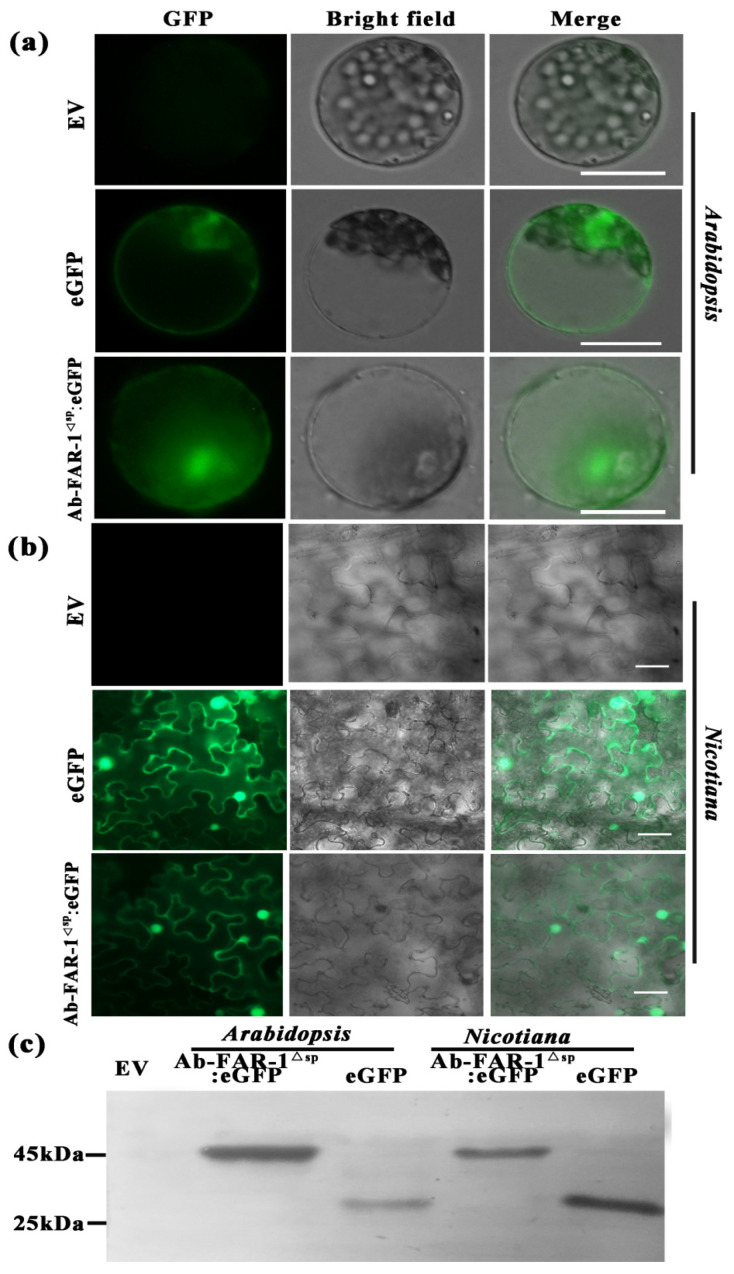
Subcellular localisation of Ab-FAR-1^Δsp^ in protoplast cells of *Arabidopsis thaliana* and leaves of *Nicotiana benthamiana.* (**a**,**b**) The fusion proteins were expressed in protoplast cells of *A. thaliana* and leaves of *N. benthamiana*. Fusion protein of Ab-FAR-1^Δsp^:eGFP and free eGFP signal were localised to the cytoplasm and nucleus. (**c**) The band size of each fusion protein was consistent with the size predicted by Western blot. EV, empty vector. Bar = 50 μm.

The localisation of Ab-FAR-1^Δsp^ in plant cells was predicted to be in the nucleus with the highest probability of 82.6% using PSORT II software [41]. To demonstrate it, eGFP-tagged Ab-FAR-1^Δsp^ was expressed in *A. thaliana* protoplasts and tobacco leaf cells, and proteins extracted from the transformed cells and tissues were analysed by Western blotting using an anti-GFP antibody. Clear green, fluorescent signals were observed in both the cytoplasm and nucleus of *A. thaliana* protoplasts and tobacco lower epidermal cells transformed with the 35S:Ab-FAR-1^Δsp^:eGFP and 35S:eGFP vectors (Figure S2 and Figure 3a,b). Additionally, the hybridising band sizes of their proteins were consistent with the predicted sizes (Figure 3c). Therefore, the fusion proteins were intact and not further modified by the plant cells. The subcellular localisation results were consistent with the predictions of the software, which indicated that Ab-FAR-1, without the signal peptide, was localised in the nucleus and cytoplasm. This might indicate that Ab-FAR-1 without a signal peptide was secreted by nematodes and acted on the cytoplasm and nucleus of the host during RWTN infection.

### 2.4. Ab-FAR-1^Δsp^ Promotes the Parasitism and Pathogenicity of RWTNs

**Figure 4:**
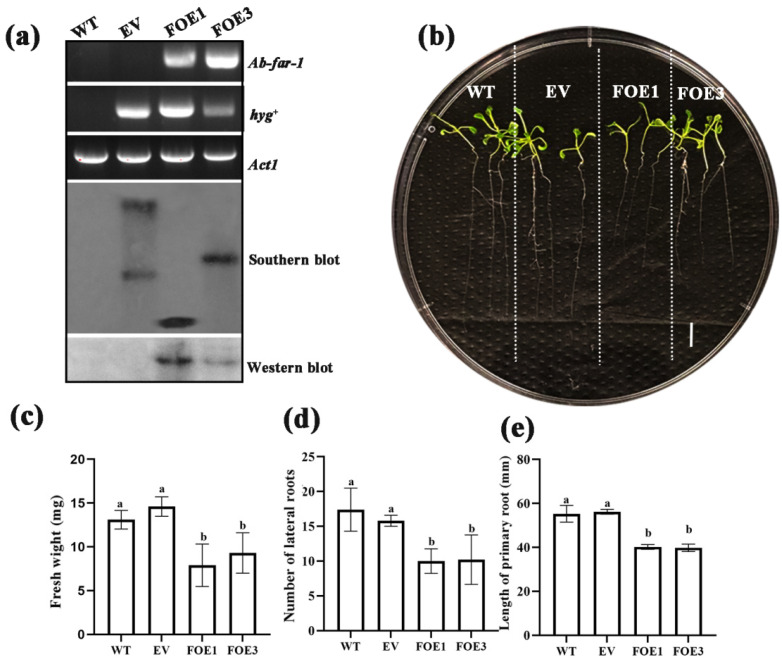
Phenotypic changes in Ab-FAR-1^Δsp^ overexpressing *Arabidopsis thaliana*. (**a**) Molecular detection of Ab-FAR-1^Δsp^ overexpressing lines. The phenotypes (**b**), fresh weight (**c**), number of lateral roots (**d**) and length of primary roots (**e**) of Ab-FAR-1^Δsp^ overexpressing lines. WT, Wild type Col-0 ecotype *A. thaliana*; EV, *A. thaliana* transformed with empty vector; FOE1 and FOE3, Ab-FAR-1^Δsp^ overexpressing *A. thaliana* line 1# (FOE1) and 3# (FOE3); *Ab-far-1*, PCR fragment bands of *Ab-far-1* gene; hyg^+^, PCR fragment bands of hygromycin resistance gene; *Act1*, reference gene *Act1*. Values are shown as means ± SE, *n* = 10. Different letters above bars denote values that are significantly different from each other (*p* < 0.05, Tukey’s test). There were three independent biological replicates. Bar = 10 mm.

To assess the role of Ab-FAR-1 in the parasitism and pathogenicity of RWTNs, we obtained two single-copy positive T3 generation homozygous overexpressing Ab-FAR-1^Δsp^ *A. thaliana* lines (FOE1 and FOE3) by transformation and screening (Figure S2 and Figure 4a). Western blot results showed that a specific hybridisation band at the expected size of ~19 kDa was detected in the transgenic *A. thaliana* lines by the anti-Ab-FAR-1B polyclonal antibody (Figure 4a), which indicated that Ab-FAR-1^Δsp^ was normally expressed in FOE1 and FOE3. Phenotypic observations of FOE1, FOE3, plants expressing the empty vector (EV), and WT plants after 10 days of growth on MS medium showed that the fresh weight, number of lateral roots and primary root length of FOE1 and FOE3 were significantly lower (*p* < 0.05) than those of WT and EV (Figure 4b–d). This result indicates that Ab-FAR-1^Δsp^ might interfere with the growth and development of *A. thaliana* and cause phenotypic changes. 

**Figure 5:**
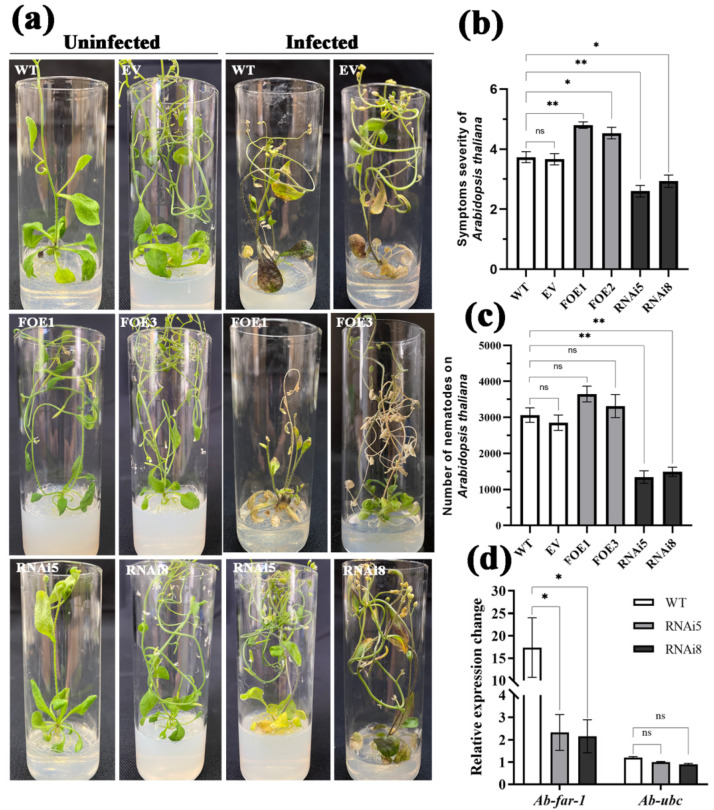
Investigation of *Aphelenchoides besseyi* parasitism on Ab-FAR-1^Δsp^ overexpressing (OE) and *Ab-far-1* silencing (RNAi) *Arabidopsis thaliana*. (**a**) Symptoms of damage in various *A. thaliana* lines. (**b**) The symptom severity in various *A. thaliana* lines. (**c**) The total numbers of nematodes were counted in various *A. thaliana* lines. These data of Ab-FAR-1^Δsp^ overexpressing line 1# (FOE1) and 3# (FOE3), *Ab-far-1* silencing line 5# (RNAi5) and 8# (RNAi8), empty vector transgenic line (EV) and their wild types (WT) were recorded at 21 days post inoculation (dpi) with *A. besseyi.* Values are indicated as the means ± SE, *n* = 15. (**d**) The *Ab-far-1* gene was silenced in *A. besseyi* collected from *A. thaliana* RNAi lines. The *Ab-18S* gene was used as an internal reference gene. The expression levels of *Ab-ubc* from *A. besseyi* were used to determine the specificity of the *Ab-far-1*-targeting RNAi. Values are means ± SE, *n* = 3. Asterisks denote significant differences (* *p* < 0.05, ** *p* < 0.01; *t*-test). There were three independent biological replicates.

Furthermore, infection assays with RWTNs showed that the symptom severity in FOE1 and FOE3 was significantly higher (*p* < 0.05) than that in WT and EV. The nematode populations on FOE1 and FOE3 were 3650 ± 217 and 3313 ± 321, respectively, which increased by 19.16% and 8.16% compared to WT (3063 ± 199), and 28.03% and 16.20% compared to EV (2851 ± 213), respectively (Figure 5a–c). Therefore, the two Ab-FAR-1^Δsp^ overexpressing lines were more susceptible to RWTN infection than the control plants, indicating that Ab-FAR-1^Δsp^ promoted the parasitism and pathogenicity of RWTNs.

### 2.5. In Planta, RNAi of Ab-far-1 Impairs the Parasitism and Pathogenicity of RWTNs

RNAi sense and antisense fragments of 130–372 bp within *Ab-far-1* were constructed into the pFGC5941 vector. Subsequently, the pFGC5941-35S: *Ab-far-1* hairpin dsRNA vector was obtained (Figure S2). Two single-copy *Ab-far-1* RNAi *A. thaliana* T3 generation lines (RNAi5 and RNAi8) were obtained through transformation and screening (Figure S3) and used for the RWTN infection assays. Two RNAi lines and WT plants were inoculated with RWTNs for 21 days, and the symptom severity was monitored. Results indicated that the symptom severity of the RNAi5 and RNAi8 lines was significantly lower (*p* < 0.05) than that of the WT (Figure 5a,b). The nematode populations on RNAi5 and RNAi8 were 1344 ± 173 and 1489 ± 130, respectively, which were significantly lower (*p* < 0.05) than those on WT (3063 ± 199) (Figure 5c). Real-time quantitative PCR results showed that the expression level of the *Ab-far-1* gene was significantly decreased by 86.65% and 87.57% upon infection of the RNAi5 and RNAi8 lines, respectively, compared with nematodes from WT (*p* < 0.05) (Figure 5d). The expression of the control gene *Ab-ubc* was not significantly different among the treatments. These results indicate that both RNAi lines specifically silenced the *Ab-far-1* gene, resulting in a decrease in the parasitism and pathogenicity of RWTNs. 

### 2.6. Ab-FAR-1 Interacts with Arabidopsis thaliana AtADF3

First, we found that Ab-FAR-1^Δsp^ could not be self-activated in the yeast self-activation assay (Figure S4). Using the Ab-FAR-1^Δsp^ protein as bait and a cDNA library derived from *A. thaliana* as prey, we performed the yeast two-hybrid (Y2H) screen of Ab-FAR-1^Δsp^. Forty independent clones were screened on high-stringency selection medium, and 21 Ab-FAR-1^Δsp^ candidate interaction genes were identified by sequencing (Figure S5; Table S1). Among them, we found a number of genes involved in the life process of *A. thaliana*, such as actin depolymerizing factor 3 (ADF3), phytochrome-associated protein 1 (PAP1), chloroplast RNA-binding protein 31B (CP31B), etc., indicating that Ab-FAR-1 may affect the host’s life activity. We found six colonies that were sequenced for the AtADF3 gene, and all of the colonies contained the complete open reading frame of AtADF3. Previous research has suggested that ADF3 may be involved in the process of pathogen–host interaction [42]. Therefore, we further studied the role of ADF3 in the interaction between RWTNs and *A*. *thaliana*.

**Figure 6:**
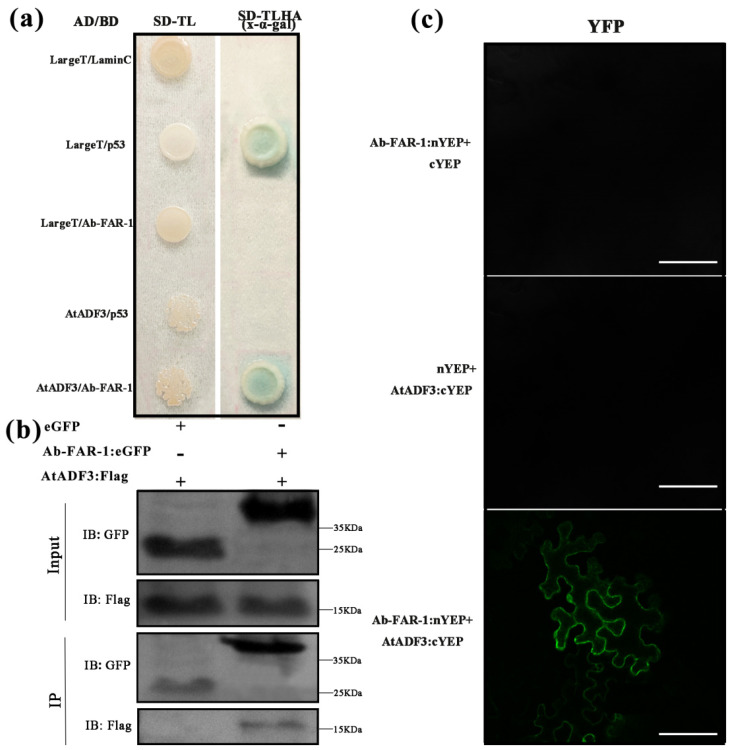
Ab-FAR-1^Δsp^ interacts with AtADF3. (**a**) The yeast two-hybrid interaction between Ab-FAR-1^Δsp^ and AtADF3. Left column, cotransformants grown on SD-TL (-trp -leu) agar medium demonstrate that both bait and prey plasmids are present in yeast (*Saccharomyces cerevisiae*); right column, only yeast cells containing the Ab-FAR-1^Δsp^ bait plus the AtADF3 prey or the positive control interaction of LargeT plus p53 grew and turned blue on the selective medium SD-TLHA (-trp –leu –his -ade) + x-α-gal agar medium. Ab-FAR-1^Δsp^, p53 and LaminC were cloned into the pGBKT7 vector, while AtADF3 and LargeT were cloned into the pGADT7 vector, respectively. LargeT was cotransformed with p53 as a positive control. LargeT/LaminC, AtADF3/LaminC and LargeT/Ab-FAR-1^Δsp^ cotransformations were negative controls. (**b**) Co-immunoprecipitation of AtADF3 with Ab-FAR-1^Δsp^. Ab-FAR-1^Δsp^:eGFP or eGFP was transiently co-expressed with AtADF3:Flag in tobacco leaf cells. The isolated protein was analysed by immunoblotting with anti-Flag antibodies to detect AtADF3 and anti-GFP antibodies to detect Ab-FAR-1^Δsp^. (**c**) Bimolecular fluorescent complementation (BiFC) visualization of the Ab-FAR-1^Δsp^ with AtADF3 interaction. Ab-FAR-1^Δsp^: nYFP and AtADF: cYFP were co-expressed in tobacco leaves. Controls were performed by co-expressing nYFP and AtADF3:cYFP, Ab-FAR-1^Δsp^:nYFP and cYFP. Bars = 100 μm as shown. Three independent experiments were performed with similar results.

It was further found that yeast AH109 co-transformed with pGBKT7:Ab-FAR-1^Δsp^ and pGADT7:AtADF3 could grow on SD/-Leu/-Trp/-Ade/-His + x-α-gal plates and turned blue (Figure 6a). To further examine this interaction, Co-IP and BiFC assays were performed. The 35S:Ab-FAR-1^Δsp^:eGFP and 35S:AtADF3:Flag vectors were co-expressed in tobacco leaf cells. Tobacco leaf cells that co-expressed 35S:eGFP and 35S:AtADF3:Flag were used as controls, and the total proteins were extracted for Co-IP analysis. Western blot analysis was used to confirm the expression of the input proteins. The hybridisation bands were at ~27, ~46, and ~16 kDa, which were consistent with the sizes of eGFP, Ab-FAR-1^Δsp^:eGFP, and AtADF3:flag, respectively. In the cells expressing 35S:Ab-FAR-1^Δsp^:eGFP and 35S:AtADF3:Flag, AtADF3:Flag was detected using an anti-Flag antibody in the sample immunoprecipitated with an anti-GFP antibody compared with control cells (Figure 6b). For BiFC analysis, 35S:Ab-FAR-1^Δsp^:nYFP and 35S:AtADF3:cYFP were co-expressed in tobacco leaf cells. The YFP fluorescent signal was detected in the cytoplasm and nucleus (Figure 6c), which corresponded to the YFP activity reconstituted by the interaction between Ab-FAR-1 and AtADF3. At the same time, no YFP signals were detected in cells expressing 35S:nYFP and 35S:AtADF3:cYFP or 35S:Ab-FAR-1^Δsp^:nYFP and 35S:cYFP (Figure 6c). Thus, both the BiFC and Co-IP assays confirmed the specific interaction between Ab-FAR-1 and AtADF3.

### 2.7. AtADF3 Is Involved in the Parasitism and Pathogenicity of RWTNs

**Figure 7:**
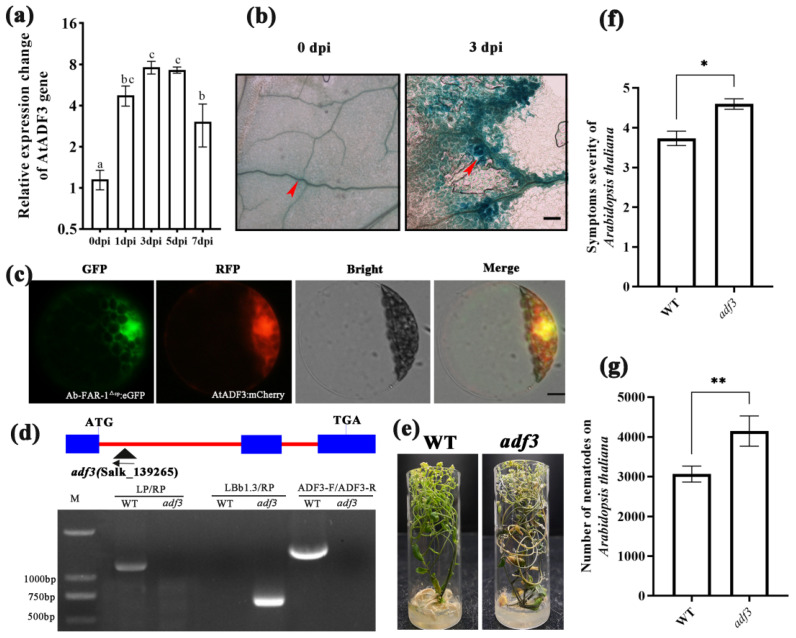
AtADF3 involved in the interaction between *Aphelenchoides besseyi* and *Arabidopsis thaliana*. (**a**) AtADF3 is upregulated in response to plant infection. Values are presented as the means ± SE, *n* = 9. (**b**) Histochemical staining for β-Glucuronidase (GUS) activity (blue colour) in nematode-infected leaves of an ADF3pro: GUS plant in which GUS expression is driven from the AtADF3 promoter. A stronger GUS signal was detected in the veins of the nematode-infected leaves than in healthy leaves. The red arrow indicates the location of the GUS signal. Bar = 200 μm. (**c**) Subcellular localization of AtADF3 in protoplast cells of *A. thaliana*. Bar = 10 μm. Different letters above bars denote values that are significantly different from each other (*p* ≤ 0.05, Tukey’s test). (**d**) *A. thaliana* lines carrying T-DNA insertion at the AtADF3 locus. Diagrammatic representation of the genomic structures of AtADF3 indicating the location of T-DNA insertions in *adf3* mutant (Salk_139265) lines relative to the translation start (ATG) and stop (TGA) codon. Exons are represented as blue boxes and introns as red lines. The direction of the T-DNA sequence is indicated by black arrow. (**e**,**f**) The symptom severity was determined in the *adf3* mutant. (**g**) The number of nematodes in the *adf3* mutant. The number of nematodes was determined after inoculation of 100 adult nematodes on each plant (including culture medium) at 21 days post inoculation. Values are presented as the means ± SE (*n* = 15). There were three independent biological replicates. Asterisks denote significant differences (* *p* < 0.05, ** *p* < 0.01; *t*-test).

After *A. thaliana* was inoculated with RWTNs, compared with uninfected *A. thaliana*, the expression of AtADF3 increased significantly from 1 dpi to 3 dpi. It then decreased from 5 dpi to 7 dpi, with the highest transcription level recorded at 3 dpi (Figure 7a). Histochemical analysis detected GUS activity in transgenic leaves expressing AtADF3pro:GUS (Figure S2). The results showed that AtADF3 was expressed in the veins of the leaves, and AtADF3 expression in RWTN-infected leaves was higher than that in healthy leaves (Figure 7b). Subcellular localisation analysis of AtADF3 showed that AtADF3 was located in the cytoplasm and nucleus of *A. thaliana* protoplasts, consistent with the localisation of Ab-FAR-1 (Figure 7c). The homozygous *A. thaliana* AtADF3 mutant (*adf3*, SALK_139265) was screened (Figure 7d) and used for the RWTN-infection assays. After 21 days, the symptom severity of *adf3* was significantly higher (*p* < 0.05) than that of the WT plants. The number of nematodes on *adf3* was also significantly increased (*p* < 0.01) by 35.27% compared with that on WT plants (Figure 7e). These results suggest that AtADF3 is involved in the interaction between *A. thaliana* and RWTNs and that the *adf3* mutant was more susceptible to RWTNs.

### 2.8. Ab-FAR-1^Δsp^ Can Affect AtADF3-Mediated Actin Filament Depolymerisation

**Figure 8:**
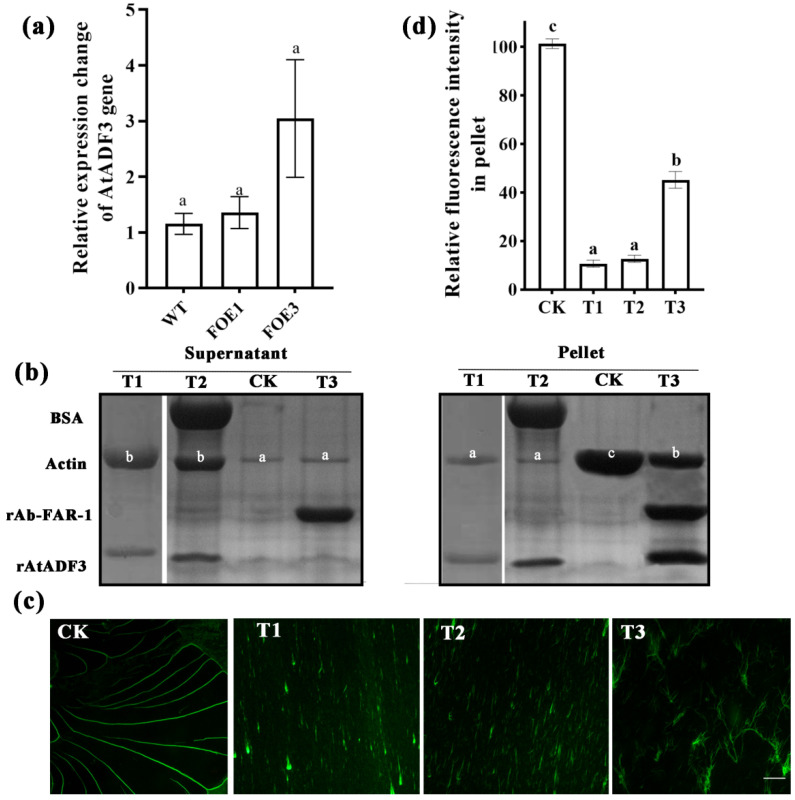
Ab-FAR-1^Δsp^ can inhibit AtADF3-mediated F-actin depolymerizing function. (**a**) Overexpression of Ab-FAR-1^Δsp^ was not affected by the transcription level of AtADF3. (**b**) The F-actin depolymerizing activities of AtADF3 were determined using high-speed co-sedimentation assays; F-actin filaments were incubated with different treatments (CK, T1-T3). The pellet and supernatant fractions were analysed by SDS–PAGE. CK, 4 μM F-actin alone; T1, 6 μM rAtADF3/ 4 μM F-actin; T2, 6 μM BSA/6 μM rAtADF3/4 μM F-actin; T3, 6 μM rAb-FAR-1/6 μM rAtADF3/4 μM F-actin. (**c**) Direct visualization of F-actin in pellet by Alexa-488-phalloidin staining method. Bar = 20 μm. (**d**) The content of actin was quantified by fluorescence intensity. There were three independent biological replicates. Different letters above bars denote values that are significantly different from each other (*p* < 0.05, Tukey’s test).

Real-time quantitative PCR results showed that there was no significant difference between the expression levels of the AtADF3 gene in *A. thaliana* overexpressing Ab-FAR-1^Δsp^ and WT *A. thaliana*, indicating that Ab-FAR-1^Δsp^ did not affect the expression of AtADF3 (Figure 8a). The recombinant AtADF3 protein (rAtADF3) was obtained by prokaryotic expression, and it was found to depolymerise actin filaments in vitro by high-speed co-sedimentation assay and fluorescence microscopy (Figure S6 and Figure 8b,c—panel T1). Then, four mixture samples (CK, T1–T3) in which rAb-FAR-1^Δsp^, rAtADF3, BSA, and F-actin were mixed in different combinations were determined by high-speed co-sedimentation assay, and the supernatants and pellets were analysed by SDS-PAGE. In the F-actin alone (CK) sample, many actin filaments were present in the pellet, and a small number in the supernatant. The actin filament content in the pellets of the rAtADF3 and F-actin mixture sample (T1) and the rAtADF3, BSA, and F-actin mixture sample (T2) was significantly lower (*p* < 0.05) than that of the CK sample, and the contents in the supernatant were significantly higher (*p* < 0.05) than that of the CK sample. The pellet and supernatant contents of the T2 sample were not significantly different from those of the T1 sample. These results indicate that rAtADF3 efficiently depolymerised F-actin in vitro and was not affected by BSA. However, the actin filament content in the pellet of the rAb-FAR-1^Δsp^, rAtADF3, and F-actin mixture sample (T3) was significantly lower (*p* < 0.05) than that of the CK sample but significantly higher (*p* < 0.05) than that of the T1 and T2 samples, and the content in the supernatant was also significantly lower than that of the T1 and T2 samples (Figure 8b). These results indicate that AtADF3 could still depolymerise F-actin, but the depolymerisation ability was inhibited when rAb-FAR-1^Δsp^ was added, indicating that rAb-FAR-1^Δsp^ could inhibit the actin depolymerisation ability of AtADF3. In addition, phalloidin staining results showed that the fluorescence intensity of the actin filaments was significantly lower (*p* < 0.05) in the pellets of the T1, T2, and T3 samples than in those of the CK sample, while the fluorescence intensity of the actin filaments in the T3 sample was significantly higher (*p* < 0.05) than that of T1 and T2. Some filamentous F-actin remained in the T3 sample (Figure 8c,d). Therefore, the interaction between Ab-FAR-1 secreted by RWTNs and AtADF3 from the host inhibited the actin filament depolymerisation function of AtADF3, which might further affect the turnover process of the actin cytoskeleton in host plant cells.

### 2.9. Actin Cytoskeleton Is Changed in Arabidopsis thaliana Overexpressing Ab-FAR-1^Δsp^

**Figure 9:**
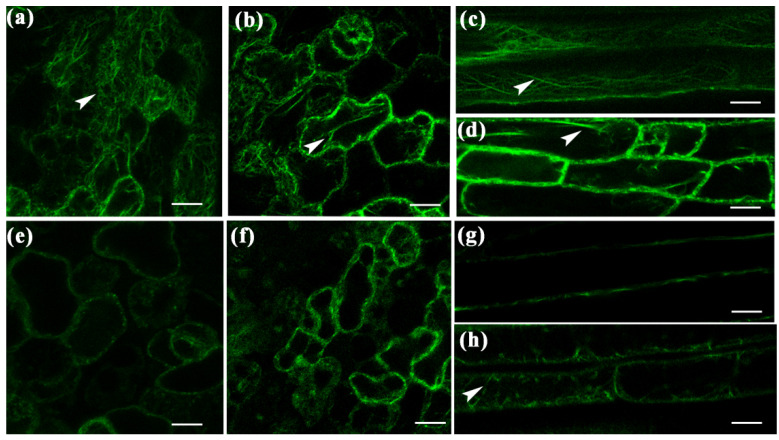
Effect of Ab-FAR-1 on actin cytoskeleton. The actin cytoskeleton (green) of leaf epidermis (**a,b**) and hypocotyl (**c,d**) in wild-type and Ab-FAR-1 transgenic wild-type *Arabidopsis thaliana* seedlings.. Latrunculin B-treated leaf epidermis (**e,f**) and hypocotyl cells (**g,h**) of wild-type and Ab-FAR-1 transgenic *A. thaliana* seedlings. The white arrow indicates the location of actin filaments. Bar = 10 μm.

The actin marker vector 35S:GFP:fABD2 (fimbrin actin-binding domain 2) [43] was transformed into *A. thaliana* overexpressing Ab-FAR-1^Δsp^ (FOE1) and WT *A. thaliana*. Subsequently, the actin cytoskeleton in 7-day-old seedlings was observed under a fluorescence microscope. In WT *A. thaliana* seedlings, the actin filaments of the leaf epidermal cells and hypocotyl cells were dense and spread throughout the whole cell (Figure 9a,c), whereas in FOE1, the actin filaments of the corresponding cells were brighter and less dense and were mainly concentrated at the cell edge (Figure 9b,d). To further investigate how Ab-FAR-1 affects the actin cytoskeleton, we compared the organisation of the actin cytoskeleton in FOE1 and control seedlings when both were treated with 5 mM Latrunculin B (LatB). LatB can prevent the polymerisation of new actin filaments from the dynamic pool of monomeric globular actin produced by the turnover of F-actin [44]. Two hours after LatB application, the actin filaments in the epidermis and hypocotyl cells of the control WT plants almost disappeared or diffused (Figure 9e,g), and residual actin filaments were still evident in the corresponding cells of FOE1 (Figure 9f,h). The effect of LatB was thus greater on WT *A. thaliana* than on FOE1, indicating that the turnover process of actin may be altered in cells of *A. thaliana* overexpressing Ab-FAR-1^Δsp^.

### 2.10. Ab-FAR-1^Δsp^ and AtADF3 Are Involved in the PTI of Arabidopsis thaliana

**Figure 10:**
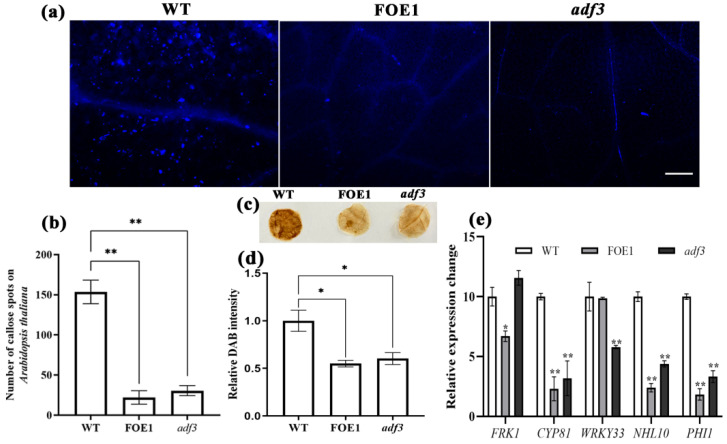
Callose deposition and ROS production was suppressed in leaves of *Arabidopsis thaliana* overexpressing Ab-FAR-1and *adf3* mutant following flg22 treatment. (**a**,**b**) The callose deposition was suppressed in leaves of *A. thaliana* overexpressing Ab-FAR-1 and *adf3* mutant following flg22 treatment. Bar=200 μm. Values are presented as the means ± SE, *n* = 15. (**c**,**d**) The ROS production was suppressed in leaves of *A. thaliana* overexpressing Ab-FAR-1 and *adf3* mutant following flg22 treatment. Values are presented as the means ± SE, *n* = 15. (**e**) The *A. thaliana* expressing Ab-FAR-1 and *adf3* mutant suppresses the expression of defence genes in *A. thaliana*. The *UBP22* was a reference gene. WT, wild-type Col-0 ecotype *A. thaliana*; FOE1, Ab-FAR-1 overexpressing *A. thaliana* line 1#; *adf3*, *A. thaliana* AtADF3 mutant. Values are presented as the means ± SE, *n* = 3. Asterisks indicate values that are significantly different (* *p* < 0.05, ** *p* < 0.01; *t*-test) from the WT.

The bacterial flagellin or its *N*-terminal peptide mimics (Flg22), as a microbe associated molecular pattern, could be recognized by the host’s FLAGELLIN-SENSING2 (FLS2) to further activate a basal resistance response PTI [45]. Hallmarks of PTI include cytosolic acidification, changes in cytoplasmic streaming patterns, rapid generation of reactive oxygen species (ROS), callose deposition, and enhanced phospholipid turnover as well as activation of mitogen-activated protein kinase (MAPK) and calcium-dependent protein kinase (CDPK) phosphorylation cascades [46]. To investigate the effects of Ab-FAR-1 and AtADF3 on plant PTI, we tested the callose deposition, ROS production, and defence gene expression levels induced by flg22. Compared with WT *A. thaliana*, callose deposition (*p* < 0.01) and ROS production (*p* < 0.05) were both significantly decreased in the leaves of *A. thaliana* overexpressing Ab-FAR-1^Δsp^ (FOE1) and *adf3* mutants following flg22 treatment (Figure 10a–d). The MAPK pathway marker genes (*FRK1*, *CYP81*, and *WRKY33*) and CDPK pathway marker genes (*NHL10* and *PHI1*) in flg22-mediated PTI of these plants were monitored using RT-qPCR (Figure 10e). The expression levels of *FRK1*, *CYP81*, *NHL10*, and *PHI1* in FOE1 and *NHL10* and *PHI1* in the *adf3* mutant were significantly down-regulated (*p* < 0.05) compared with those in WT *A. thaliana*. These results suggest that Ab-FAR-1^Δsp^ and AtADF3 are both involved in the MAPK and CDPK pathways of plant immunity, and they might have overlapping functions, which indicates that Ab-FAR-1^Δsp^ might further suppress *A. thaliana* PTI through AtADF3. 

## 3. Discussion

Fatty acid and retinol binding proteins, which are nematode-specific proteins, are mainly responsible for assisting nematodes in obtaining essential fatty acids and retinols. Many studies have also revealed their potential role in nematodes’ host infection and pathogenicity, acquired resistance, and immune regulation [30,34]. In previous studies, eight *Ab-far* genes were identified in RWTN, but their functions vary. Among these, *Ab-far-1* has been shown to probably be involved in the reproduction and pathogenic processes of nematodes [26,39,47]. However, the detailed functions and mechanisms of *Ab-far-1* in the parasitism and pathogenicity process of nematodes have not been studied. In this study, the role and mechanism of *Ab-far-1* in RWTN parasitism were studied in the model plant *A. thaliana*. We discovered a novel mechanism of Ab-FAR-1, which interacts with *A. thaliana* AtADF3 to alter and regulate the state and turnover process of cellular actin filament networks, thereby promoting nematode parasitism and pathogenicity.

Proteins secreted by the oesophageal glands and body walls of parasitic nematodes are considered the key factors that regulate the interaction of the nematodes with their hosts and thus contribute to their parasitism and pathogenesis [11,21,48]. Protein Ab-FAR-1 has a secretory signal peptide at the *N*-terminal, and its mRNA is present in the hypodermis of juvenile and adult nematodes and the gonads of adult nematodes [26]. The current study further confirmed that the Ab-FAR-1^Δsp^ protein was mainly located in the body wall of nematodes and in the cytoplasm and nucleus of host cells by using immunofluorescence and subcellular localisation. The cytoplasm and nucleus are the main functional sites of many nematode effector proteins [18]. Therefore, during parasitism by RWTN, the Ab-FAR-1 protein is secreted into the host through the body wall of the nematodes and might act on the cytoplasm and nucleus of the host cells, which in turn, might play important regulatory roles in the host defence response. However, how RWTN-secreted Ab-FAR-1 crosses the cell membrane and enters the cells of the host may require further study. 

To test the role of Ab-FAR-1 in RWTN parasitism, transgenic *A. thaliana* lines overexpressing Ab-FAR-1^Δsp^ were generated. Overexpression of Ab-FAR-1^Δsp^ interfered with the growth and development of *A. thaliana* and caused thinner plants and shorter roots than those of WT *A. thaliana*. Previous studies have reported that overexpression of some nematode effector proteins in plants can cause phenotypic changes. For example, the lengths of the primary roots are elongated in rice overexpressing *M. graminicola* MgMO237 [18], and overexpression of *Meloidogyne enterolobii* MeTCTP causes reduction of lateral roots in *A. thaliana* [49]. It is speculated that the changes in the root phenotype may be due to the effector proteins secreted by nematodes causing metabolic disorders within the plant host cells. To further validate the virulence function in nematode parasitism, *Ab-far-1* was silenced using *in planta* RNAi. Compared with WT *A. thaliana,* transgenic lines overexpressing Ab-FAR-1^Δsp^ were more susceptible to nematode infection, whereas *Ab-far-1* RNAi lines showed the opposite trend. Therefore, Ab-FAR-1 may play a role in the parasitism and pathogenicity of RWTN.

In recent years, the discovery of host proteins that interact with nematode-secreted effectors has led to the increasingly specific elucidation of the pathogenic mechanisms of parasitic nematodes. *M. graminicola* MgMO289 was recently discovered and demonstrated to promote nematode parasitism by utilising the host O_2_^•−^-scavenging system to eliminate O_2_^•−^ and suppress plant immunity [50]. *M. incognita* MiMIF-2 can interact with the two annexins of *A. thaliana* and damage Ca^2+^ signal transduction to interfere with plant immunity [51]. The current study demonstrated that Ab-FAR-1 interacts with AtADF3 in *A. thaliana* and that this interaction occurs in the cytoplasm and nucleus of the plant cells. *A. thaliana* ADF3 expression is the strongest among the ADF family of genes in *A. thaliana* [52]. Gene expression data available in the *Arabidopsis* eFP Browser (http://bbc.botany.utoronto.ca/efp/cgi-bin/efpWeb.cgi, accessed on 20 July 2022) revealed that ADF3 is expressed throughout the development of *A. thaliana* in most organs, except pollen. Our results showed that the expression level of AtADF3 in leaf veins of *A. thaliana* infected with RWTN was higher than that of healthy *A. thaliana*, which indicated that AtADF3 might be involved in the nematode infection process. Phloem, duct, and sieve tubes accumulate in the veins of plants and contain a large amount of water and nutrients that can be used by nematodes during feeding. According to the literature, the actin filament turnover process mediated by the ADF protein family is involved in the interaction between the host and pathogens such as nematodes, bacteria, viruses, and fungi [9]. *A. thaliana adf4* mutants were more susceptible to the DC3000 population of *Pseudomonas syringae* pv. Tomato (*Pst*) because actin remodelling and single-filament dynamics in *adf4* are insensitive to the peptide mimics (elf26) of bacterial elongation factor Tu treatment [7,8]. The RPS5-AvrPphB-mediated ETI signal transduction pathway in *A. thaliana* requires phosphorylation and dephosphorylation of AtADF4 to regulate the participation of dynamic rearrangements of the cell actin network [7]. In addition, the *Soybean mosaic virus*-encoded P3 protein can directly interact with soybean ADF2, promoting movement and replication along actin microfilaments of the P3 protein and the spread of the virus [53]. Additionally, *A. thaliana* AtADF3 mutants are more susceptible to aphids, which may be related to the loss of AtADF3-mediated actin filament depolymerisation [42]. In contrast, AtADF2 knockout inhibits root-knot nematode parasitism [54,55], and knockout of AtADF4 enhances resistance to powdery mildew fungi [56]. Therefore, different ADF proteins play different roles in plant defences or susceptibility to pathogens. In this study, compared with WT *A. thaliana*, the symptoms were significantly aggravated and the number of nematodes was significantly increased after RWTN infection in the *adf3* mutant. Thus, Ab-FAR-1 secreted by RWTNs may promote parasitism and pathogenicity of nematodes by interacting with the AtADF3 protein.

It has been reported that cytoskeletal rearrangement of actin filaments is necessary for the development of giant cells formed in hosts infected by root-knot nematodes [57]. Knockout of AtADF2 stabilises the actin cytoskeleton, which prevents normal giant cell maturation in root-knot nematodes and leads to a significant reduction in the number of nematode invasions [55]. In general, ADFs bind to both monomeric globular (G-) and filamentous (F-) actin to increase actin dynamics by severing microfilaments and increasing the dissociation rate of actin monomers [58,59,60]. In this study, we found that the interaction between rAb-FAR-1^Δsp^ and rAtADF3 inhibits the function of AtADF3 in depolymerising F-actin. The state, distribution, and turnover of the actin cytoskeleton were altered in *A. thaliana* overexpressing Ab-FAR-1^Δsp^. Therefore, RWTNs may affect the function of AtADF3 by secreting Ab-FAR-1, which indirectly affects the actin cytoskeleton and promotes the pathogenic process of RWTNs. Results also indicated that the plant actin cytoskeleton could be used as an important target to prevent nematode infection or as an important structure of plant defences against nematode infection. 

In recent years, the actin cytoskeleton has been shown to play a key role in plant immune signalling. The actin cytoskeleton can facilitate immune signalling, playing roles in the maintenance, surveillance, activation, execution, and possibly de-escalation of plant immunity [6]. The actin-depolymerizing factor proteins are important for actin turnover, and play diverse roles in plant immunity. For example, the localisation of AtADF4 to the nucleus is related to the decreased expression of the PTI defence gene *FRK1* in *A. thaliana* [61]. Additionally, the *Pst* PAMP elf26 elicitor can directly increase the abundance of actin filaments in *A. thaliana* cells, which is inhibited when AtADF4 is knocked out [8,61,62]. These results directly prove that the turnover of the actin cytoskeleton can be used as a marker, and that the ADF protein family may play key regulatory roles in the PTI of plants. In addition, it has been reported that both *A. thaliana adf3* and *adf4* mutants can inhibit the cell wall-strengthening process of callose deposition [8,42]. Studies on the function and mechanism of FARs in the inhibition of plant defences have been reported. The potato cyst nematode *Globodera pallida* Gp-FAR-1 binds to two lipids (linolenic and linoleic acids), which are precursors of plant defence compounds and the jasmonic acid signalling pathway [29]. Protein Mj-FAR-1 produced by the root-knot nematode *M. javanica* regulates the expression of lipid-, cell wall-, stress-, and phenylpropanoid-related genes during nematode infection in tomatoes, as determined by transcriptome analysis [31]. Protein Bx-FAR-1 secreted by the pine wood nematode *Bursaphelenchus xylophilus*, interacts with the F-box protein of pines to mediate the jasmonic acid pathway and facilitate parasitism of the nematodes in pines [24]. In this study, the process of callose deposition and ROS bursts and the transcriptional activation of two genes *PHI1* and *NHL10* of CDPK pathway and *CYP81* gene of MAPK pathway were both inhibited in *A. thaliana* overexpressing Ab-FAR-1^Δsp^ and *adf3* mutant after flg22 treatment. These results showed that Ab-FAR-1^Δsp^ and AtADF3 were involved in PTI immune signal transduction in the plants. In addition, the *FRK1* gene was also inhibited in *A. thaliana* overexpressing Ab-FAR-1^Δsp^, but not in the *adf3* mutant, indicating that Ab-FAR-1 has other complex pathways and functions in plant defence responses.

In conclusion, Ab-FAR-1 may not only have the previously reported function of helping nematodes to obtain fatty acids and retinols but may also affect the distribution and turnover of actin filaments in plants by interacting with AtADF3, suppressing the plant PTI, and promoting the parasitism of RWTNs. In addition, the current study also enriches our understanding of the relationship between the actin cytoskeleton and defence responses in plants. The knockout of AtADF2 affects the development of giant cells and inhibits the infection of root-knot nematodes [55]. This study confirmed that the knockout AtADF2 was more susceptible to RWTNs. Whether these results are related to the different parasitic pathways to hosts of sedentary and migratory plant-parasitic nematodes is an interesting question. Therefore, further studies on the function of FAR and the relationship between the actin cytoskeleton, nematode parasitism, and pathogenicity may be of great significance for understanding and controlling different plant-parasitic nematodes. 

## 4. Materials and Methods

### 4.1. Biological Material and Culture 

The RWTNs used in this study were collected, isolated, and identified by the Plant Nematology Laboratory of the South China Agricultural University. Nematodes were cultured and preserved on wild-type (WT) *Botrytis cinerea* in Petri dishes (diameter 6 cm) at 25 °C [26]. The seeds of Col-0 ecotype *A. thaliana* were preserved in our laboratory. Surface-sterilised seeds of *A. thaliana* (including WT, transgenic lines, and mutants) were seeded and cultured in Murashige and Skoog (MS) medium (Sigma-Aldrich, St. Louis, MO, USA), as previously described [63]. *Nicotiana benthamiana* seeds were grown in pots mixed with common compost and sand (4:1) at 23 °C, 60–75% relative humidity, light for 16 h, and dark for 8 h in an incubator. One hundred mixed-stage nematodes were inoculated into the leaves of *A. thaliana*. The symptoms in *A. thaliana* caused by RWTN infection were monitored, and nematodes were extracted from *A. thaliana* 21 days after inoculation. The ratings of symptom severity on *A. thaliana* caused by foliar nematodes were assigned as follows [63]: rated 0 = no lesion/chlorosis, 1 = 10% lesion/chlorosis, 2 = 11–25% lesion/chlorosis, 3 = 26–50% lesion/chlorosis, 4 = 51–75% lesion/chlorosis, 5 = 75% or more lesion/chlorosis. Each treatment was replicated five times, and each experiment was conducted in triplicate to confirm the results. The method of extracting nematodes from *A. thaliana* was performed according to the reference [63].

### 4.2. RNA Extraction and Vector Construction

Total RNA was extracted from nematodes and plants using the RNeasy Micro RNA extraction kit (Qiagen, Hilden, Germany) and TRIzol reagent (Invitrogen, Carlsbad, CA, USA). The RNA was used as a template for cDNA synthesis using the ReverTra Ace RT-qPCR kit (Toyobo, Osaka, Japan). The vectors and vector construction processes are shown in Figure S2, and primers for gene fragments and PCR detection are shown in Table S2. Vector ligation was performed using an In-Fusion^®^ HD Cloning Kit (Takara, Shiga, Japan). The ligation products were transformed into *Escherichia coli* DH5α and verified by sequencing at Shanghai Sangong Co., Ltd. (Sangong, Shanghai, China).

### 4.3. Generation of Transgenic Arabidopsis thaliana

According to the floral dipping method previously described [64], the constructed vectors pCAMBIA1300-35S:Ab-FAR-1^Δsp^ and pFGC5941-35S:*Ab-far-1* hairpin RNA (Figure S1) were transformed into WT *A. thaliana*. Subsequently, overexpressing Ab-FAR-1 without a signal peptide (Ab-FAR-1^Δsp^) (FOE) and *Ab-far-1* RNAi *A. thaliana* (RNAi) were obtained. Two single copies of homozygous lines of T3 generation (FOE1 and FOE3; RNAi5 and RNAi8) were obtained through screening and culture, RT-PCR identification of resistance genes, and inserted fragments, Southern blot, and Western blot experiments, respectively. The AtADF3 T-DNA mutant of *A. thaliana* (*adf3* mutant, SALK_139265) was purchased from the Arashare platform (https://www.arashare.cn, accessed on 1 July 2021), and the homozygous *adf3* mutant was screened and obtained using three primers (LP, RP, and LBb1.3).

### 4.4. Protein Prokaryotic Expression and anti-Ab-FAR-1^Δsp^ Polyclonal Serum Preparation

The vectors pET32a:Ab-FAR-1^Δsp^ and pET28a:AtADF3 were transformed into *E. coli* BL21(DE3) for prokaryotic expression and purification, and the recombinant Ab-FAR-1^Δsp^ (rAb-FAR-1^Δsp^) and AtADF3 (rAtADF3) proteins were obtained [32,65]. The concentration and purity of the proteins were determined using the BCA method (Tiangen Biotechnology, Beijing, China) and sodium dodecyl sulphate polyacrylamide gel electrophoresis (SDS-PAGE). Anti-Ab-FAR-1^Δsp^ polyclonal serum was obtained via rabbit immunisation. The effectiveness of the Anti-Ab-FAR-1^Δsp^ antibody was determined using ELISA and Western blotting.

### 4.5. Immunofluorescence Localisation of Ab-FAR-1^Δsp^

The newly propagated adult nematodes were collected and soaked in phosphate-buffered saline (PBS) containing 2% paraformaldehyde for 18 h at 4 °C, and for 4 h at room temperature. Then, immunofluorescence localisation of the nematode bodies was performed according to the method previously described [66]. For immunofluorescence localisation of plant tissue sections, the carrot callus [67] at 3 days post-infection (dpi) with RWTNs was dissected, fixed, dehydrated, paraffin-embedded, and sectioned as described previously [68]. Then, the sections were treated with 100 μL 1:100 diluted anti-Ab-FAR-1B primary antibody at 4 °C overnight incubation in a humid box. They were then washed three times for 5 min using PBS and then incubated with 1:1000 diluted goat anti-rabbit secondary antibody conjugated to Alexa Fluor 594 (Thermo Fisher Scientific, Waltham, MA, USA), at room temperature for 2 h in a humid box. Finally, the sections were washed thrice with PBS and incubated with Fluoromount-G (SouthernBiotech, Cambridge, UK) containing DAPI for 5 min at room temperature. Immunofluorescence localisation slides of nematode bodies and plant tissues were observed and photographed under a Nikon ECLIPSE 90i fluorescence microscope (Nikon, Minato, Japan).

### 4.6. Subcellular Localisation of Ab-FAR-1^Δsp^

The vectors pCAMBIA1300-35S:Ab-FAR-1^Δsp^:eGFP, pCAMBIA1300-35S:AtADF3:mCherry, and pCAMBIA1300-35S:eGFP (control) were constructed for subcellular localisation and stored after sequencing verification (Figure S2). Each plasmid was transformed into *Agrobacterium tumefaciens* strain GV3101 and transiently expressed in 4-week-old *N. benthamiana* (tobacco leaves) for 48 h, as described previously [17]. Leaf protoplasts of *A. thaliana* were prepared and transformed, as described previously [69]. Then, the protoplasts were transferred into multi-well plates and incubated at 23 °C for 18 h. Finally, the tobacco leaves and *A. thaliana* protoplasts were examined under a Nikon ECLIPSE 90i microscope (Nikon). Total proteins from 1 × 10^6^ protoplasts and 1 g tobacco leaves were extracted separately using RIPA lysis buffer (50 mM Tris-HCl, 2% sodium dodecylsulfate (SDS), 0.1% bromophenol blue, 10% glycerin, 1% β-mercaptoethanol) and (2% SDS, 80 mM Tris/HCl, pH 6.8, 10% glycerol, 0.002% bromophenol blue, 5% β-mercaptoethanol, and a complete protease inhibitor cocktail) [49]. Western blot analysis was performed to verify intact fusion proteins. The experiment was repeated thrice.

### 4.7. Interaction Analysis

The Ab-FAR-1^Δsp^ sequence was cloned into the pGBKT7 vector to generate pGBKT7:Ab-FAR-1^Δsp^ and transformed into *Saccharomyces cerevisiae* strain AH109 as the bait strain. An *A. thaliana* cDNA library was generated from *S. cerevisiae* strain Y187. Yeast two-hybrid (Y2H) screening was performed according to the Matchmaker Gold Yeast Two-Hybrid System Kit User Manual (Clontech, Tokyo, Japan). Then, according to the Yeastmaker Yeast Transformation System 2 User Manual (Clontech), the bait and prey vectors were co-transformed with the *S. cerevisiae* strain Y2HGOLD. The α-galactosidase (MEL1) reporter gene was used to confirm this interaction in the presence of 5-bromo-4-chloro-3-indolyl-α-D-galactopyranoside (X-α-gal, Takara).

For co-immunoprecipitation (Co-IP) assays, the constructed vectors pCAMBIA1300-35S: Ab-FAR-1^Δsp^:eGFP and pCAMBIA1300-35S:AtADF3:Flag (Figure S2) were co-expressed using the *Agrobacterium*-mediated method in tobacco leaves. Tobacco leaves were co-expressed with pCAMBIA1300-35S:eGFP, and pCAMBIA1300-35S:AtADF3:Flag was used as a control. After 48 h, total leaf proteins were extracted, and the Co-IP experiment was carried out according to the instructions of the Capturem^TM^ IP & Co-IP Kit (Takara). 

For bimolecular fluorescent complementation (BiFC) assays, the constructed vectors pSPYNE-35S:Ab-FAR-1^Δsp^:nYFP and pSPYCE-35S:AtADF3:cYFP (Figure S2) were co-expressed in tobacco leaves using the *Agrobacterium*-mediated method. Tobacco leaves transiently co-expressing pSPYNE-35S:Ab-FAR-1^Δsp^:nYFP/pSPYCE-35S:cYFP and pSPYNE-35S:nYFP/pSPYCE-35S:AtADF3:cYFP were used as controls. After 48 h, reconstructed YFP signals were observed under the GFP channel of a Nikon ECLIPSE 90i microscope (Nikon).

### 4.8. β-Glucuronidase (GUS) Staining

A 2035 bp DNA fragment upstream of the transcriptional start site of AtADF3 was amplified using *Arabidopsis* genomic DNA as a template and ADF3pro-GUS-F/ADFpro-GUS-R primers (Table S2). The PCR product was cloned into pCAMBIA1381Z to obtain the pCAMBIA1381Z:AtADF3pro:GUS vector containing the coding sequence of the bacterial *uidA* gene, which encodes the GUS protein [46]. Transgenic plants containing the ADF3pro:GUS construct were generated from a background of WT *A. thaliana*. Using 5-bromo-4-chloro-3-indolyl-b-D-glucopyranosiduronic acid (X-gluc) as the substrate (Zhongkelitai Biotechnology, Beijing, China), the histochemical analysis of GUS activity was performed in the leaves of 2-week-old transgenic *A. thaliana* inoculated with RWTNs after 3 days.

### 4.9. Ab-FAR-1 Affects the Ability of AtADF3 to Depolymerize Actin Filaments

Actin was purified from rabbit skeletal muscle acetone powder and polymerised to filamentous actin (F-actin) [70]. The tested proteins were mixed into four reaction groups: 4 µM polymerised F-actin alone (CK); 6 μM rAtADF3 and 4 µM polymerised F-actin (T1); 6 μM BSA, 6 μM rAtADF3, and 4 µM polymerised F-actin (T2); and 6 μM rAb-FAR-1^Δsp^, 6 μM rAtADF3, and 4 µM polymerised F-actin (T3). Then, 200 µL buffer A3 (10 mM Tris-HCl, 0.2 mM CaCl_2_, 0.2 mM ATP, 0.5 mM DTT, pH = 8.0) was added to each reaction group and incubated at 22 °C for 1.5 h. A high-speed co-sedimentation assay was used to evaluate the F-actin depolymerisation activity of each group, as described previously [71]. Briefly, the reaction mixture of each group was co-sedimented (100,000× *g* for 30 min at 4 °C) using a high-speed centrifuge (Beckman, Brea, CA, USA). Then, 160 µL of the supernatant was transferred to a newly prepared tube, and the remaining liquid was aspirated. Thereafter, 40 µL of 10× protein loading buffer was added to the supernatant, and 200 µL of 2× protein loading buffer was added to the pellet. Finally, the supernatant and pellet were boiled for 5 min, and 20 µL was loaded and analysed separately by SDS-PAGE. The gels were stained with Coomassie Brilliant Blue Staining Solution (Sigma-Aldrich). Photographs were taken after decolourisation, and the relative actin content was quantified using the Gray value of the bands. Fluorescence microscopy was used to directly visualise the effects of the mixed recombinant proteins on the organisation of actin filaments, as previously described [65]. This experiment was repeated thrice.

### 4.10. Changes in Actin Cytoskeleton of Arabidopsis thaliana Overexpressing Ab-FAR-^Δsp^

To observe the changes in the actin cytoskeleton of *A. thaliana* ectopically overexpressing Ab-FAR-1^Δsp^, a WT *A. thaliana* (as a control) and transgenic *A. thaliana* overexpressing Ab-FAR-1^Δsp^ (harbouring an eGFP fused to fABD2 [43] under the control of the 35S promoter (pCAMBIA1300-35S:eGFP:fABD2, Figure S2)) were generated. These seedlings were treated with 5 mM latrunculin B (LatB) (Merck, Darmstadt, Germany) and DMSO solution as a control for 2 h, and the actin cytoskeletons in leaf cells and hypocotyl cells were observed using a Nikon A1 fluorescence confocal microscope (Nikon).

### 4.11. PTI Assay

To determine callose deposition, ROS production, and defence gene expression levels, 4-week-old leaves of WT, Ab-FAR-1^Δsp^ ectopically overexpressing line 1 (FOE1), and *adf3* mutant *A. thaliana* were infiltrated in 0.01 M PBS buffer (pH 5.8) containing 10 μM flg22. After 1 h, the leaf discs from the infiltrated site were removed with a punch, and the defence gene expression levels (*FRK1*, *CYP81*, *WRKY33*, *NHL10*, and *PHI1*) were monitored by RT-qPCR. ROS production was analysed by DAB (Sigma-Aldrich) staining after 24 h, as described previously [72]. The leaf discs were removed again, and callose deposition was detected by aniline blue (Sigma-Aldrich) staining, as described previously [73]. Finally, ROS production and callose deposition were observed using a Nikon ECLIPSE 90i microscope and quantitatively analysed using the ImageJ 1.48v software [74]. At least five leaf discs from each treatment were selected for quantitative analysis, and each experiment was repeated in triplicate.

### 4.12. Western Blot 

Western blot analysis was performed with 1:5000 diluted primary antibodies (anti-Flag and anti-GFP; Proteintech, Chicago, IL, USA) and 1:5000 diluted goat anti-mouse IgG and HRP conjugate (Proteintech) as described previously [75]. The proteins were visualized using DAB Enhanced substrate kit (Solarbio, Beijing, China). 

### 4.13. Real-Time Quantitative PCR (RT-qPCR) 

Real-time quantitative PCR of RWTNs and *A. thaliana* genes was performed using the primers of each gene shown in Table S2 and the instructions of SYBR qPCR Mix (Toyobo) and the CFX96^TM^ Real-time System (Bio-Rad, Hercules, CA, USA). *A. thaliana* ubiquitin carboxyl terminal hydrolase 22 (*UBP22*) and RWTN *18S* were chosen as the reference genes. The relative changes in gene expression were calculated using the 2^-ΔΔCT^ method [76]. Each sample reaction was run in triplicate.

### 4.14. Data Analyses

All data were verified by three independent experiments and shown as means ± standard error (SE). One-way ANOVA was performed using GraphPad Prism 9, and multiple comparisons were performed using Tukey’s test, with a significance level of *p* < 0.05. The *t*-test method was used to compare the experimental group with the control group to determine whether there was significant difference (* *p* < 0.05, ** *p* < 0.01) in the mean values. 

## Data Availability

Data sharing is not applicable to this article as no new data were created or analyzed in this study.

## Supplementary Materials

The supporting information can be downloaded at: https://www.mdpi.com/article/10.3390/ijms232012280/s1.
