# Supplementary material for: Aphelenchoides besseyi Ab-FAR-1 Interacts with Arabidopsis thaliana AtADF3 to Interfere with Actin Cytoskeleton, and Promotes Nematode Parasitism and Pathogenicity
Source: Int J Mol Sci. 2022 Oct 14;23(20):12280. doi: 10.3390/ijms232012280 (PMC9603084; doi:10.3390/ijms232012280)
Supplement: Supplementary file 1 [file ijms-23-12280-s001.zip › ijms-1853538-supplementary.pdf]

(a)

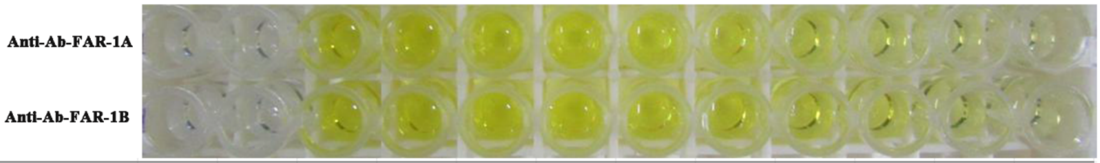

	Negative	Blank	1K	2K	4K	8K	16K	32K	64K	128K	256K	512K
Anti-Ab-FAR-1A	0.064	0.046	1.15	1.179	1.056	1.012	0.917	0.732	0.545	0.401	0.267	0.222
Anti-Ab-FAR-1B	0.043	0.052	1.135	1.146	1.157	1.136	1.017	0.875	0.636	0.459	0.371	0.243

(b)

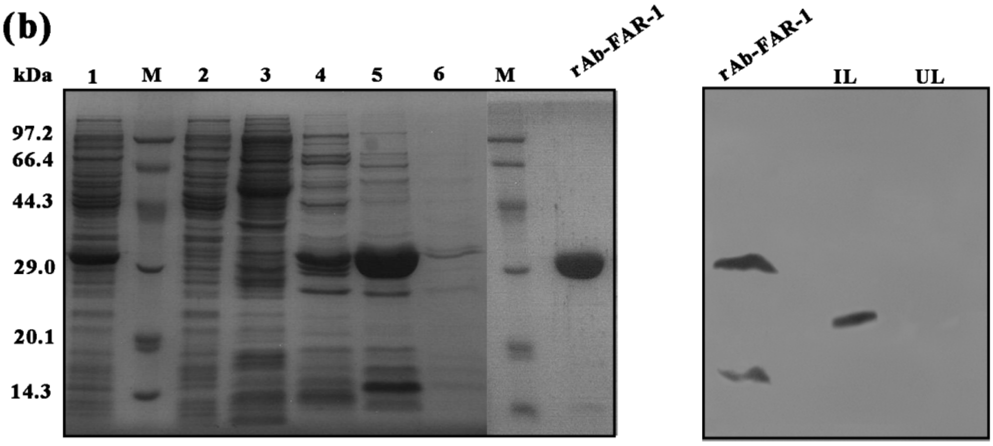

Figure S1 Validity detection of Anti-Ab-FAR-1. (a) ELISA test results and data, antibody potency value ≥ 2.5 × the negative value, the antibody potency value of Anti-Ab-FAR-1A and Anti-Ab-FAR-1B were greater than 512K; (b) The results of SDS-PAGE and Western blot analysis, SDS-PAGE (left panel) identified proteins from recombinant Ab-FAR-1$^{\triangle sp}$ of prokaryotic expression (rAb-FAR-1$^{\triangle sp}$). M, Protein Marker; 1, Load sample; 2, Flow through; 3-6, Elution by 20 mM, 50 mM, 200 mM and 500 mM imidazole. Western blot analysis (right) the Ab-FAR-1 protein of *Arabidopsis thaliana* leaves infected (IL) and uninfected nematodes (UL).

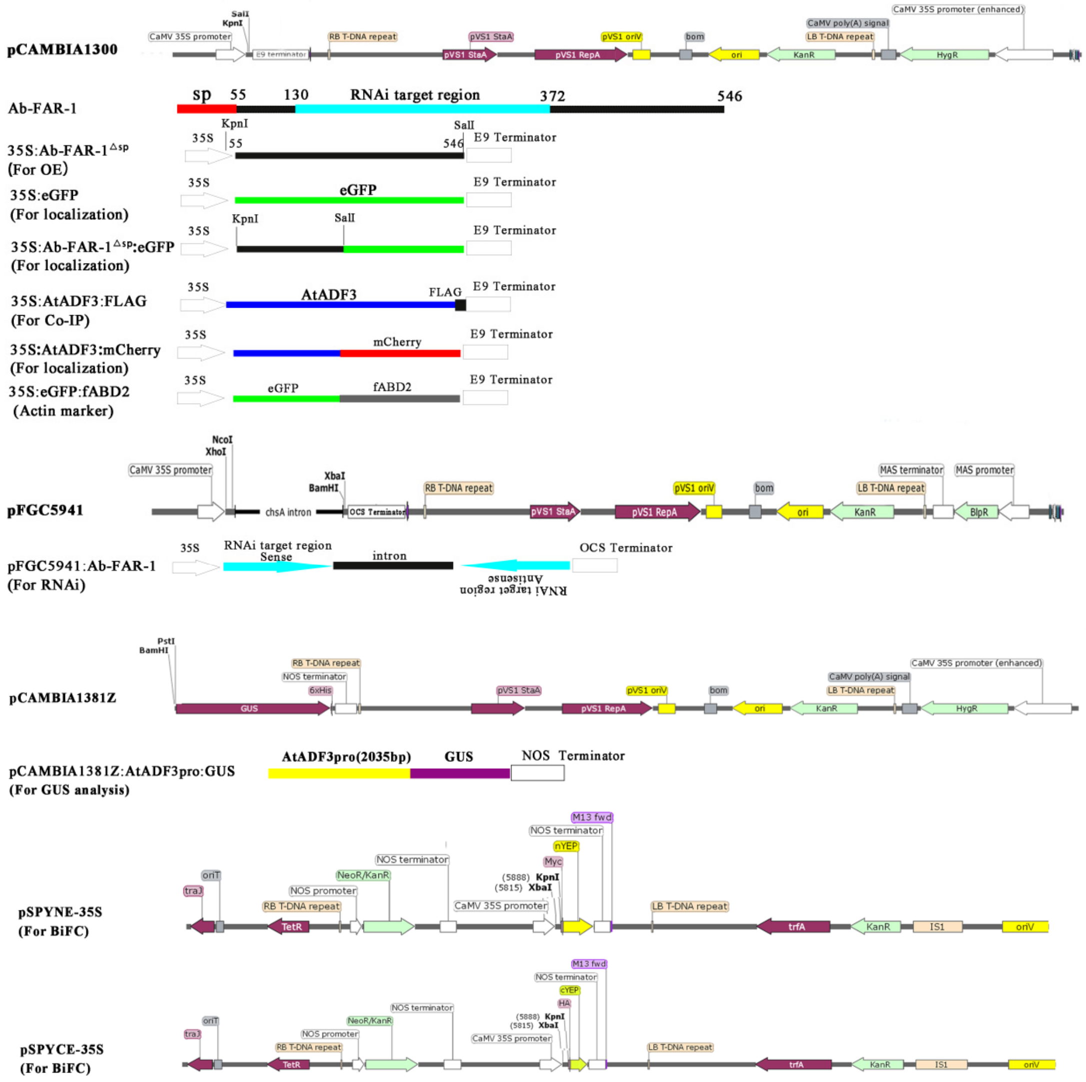

Figure S2 Schematic diagram of vector construction in this study.

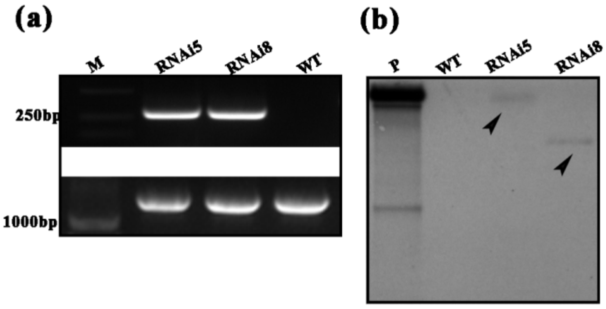

Figure S3 Molecular detection of *Ab-far-1* RNAi *Arabidopsis thaliana*. (a) Real-time quantitative PCR detection of *Ab-far-1* RNAi lines. (b) Southern blot of *Ab-far-1* RNAi lines. WT, Wild type Col-0 ecotype *A. thaliana*; RNAi5 and RNAi 8, *Ab-far-1* RNAi line 5# and 8#.

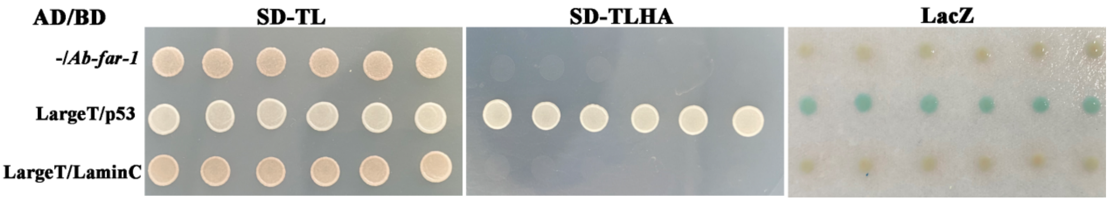

Figure S4 Self-activation detection of Ab-FAR-1$^{\triangle sp}$. pGADT7:LargeT/pGBKT7:p53, positive control; pGADT7:LargeT/pGBKT7:LaminC: negative control.

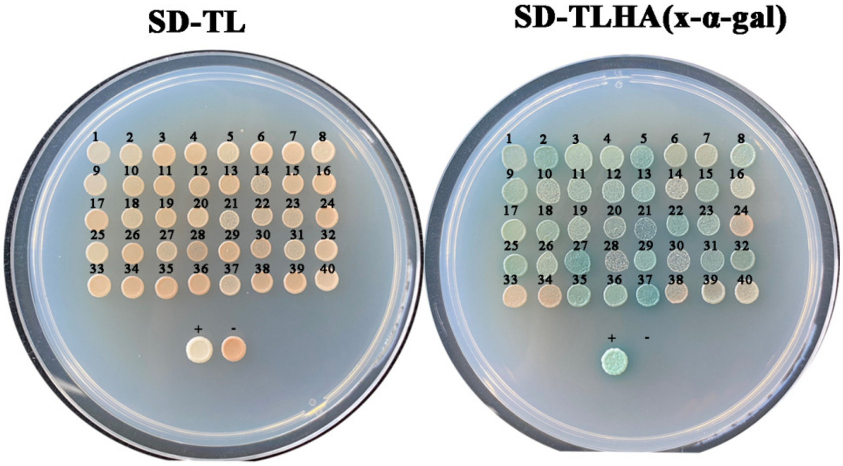

Figure S5 HIS3, ADE2 and MEL1 reporter gene detection of positive colonies in the screening library. All 40 positive colonies can activate the HIS3 and ADE2 reporter genes. Among them, 34 colonies can activate the MEL1 reporter gene. Colonies 24, 28, 30, 33, 34 and 38 cannot activate MEL1. See Table S1 for the sequence information of all colonies.

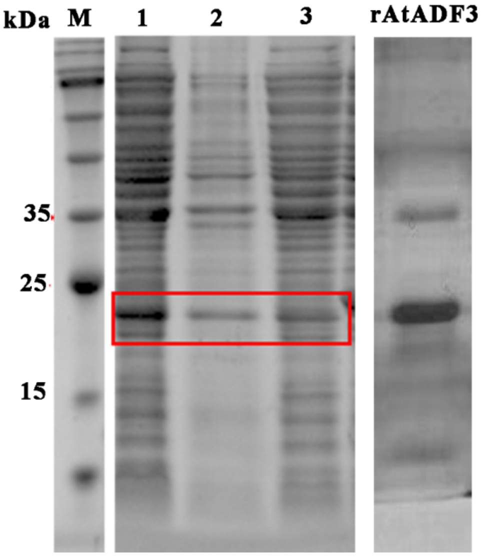

Figure S6 SDS-PAGE identified proteins from recombinant AtADF3 of prokaryotic expression (rAtADF3). M, Protein Marker; 1, crushing bacterial liquid; 2, the proteins in precipitation; 3, the proteins in the supernatant; rAtADF3, the protein purified from supernatant.

Table S1 Candidate proteins interact with Ab-FAR-1 that identified by Y2H.

Codes	NCBI Accession	NCBI Description
1, 5, 18, 21, 22, 25	NM_180896.3	Arabidopsis thaliana actin depolymerizing factor 3 (ADF3), mRNA
2	NM_001334174.1	Arabidopsis thaliana branchless trichome (BLT), mRNA
3, 4, 9, 10, 11, 12, 13, 19, 23, 26, 28, 35, 36	NM_112521.4	Arabidopsis thaliana phytochrome-associated protein 1 (PAP1), mRNA
6	NM_120280.4	Arabidopsis thaliana E3 ubiquitin-protein ligase RLIM-like protein (SIS), mRNA
7	NM_125689.5	Arabidopsis thaliana RNA polymerase II, Rpb4, core protein (AT5G62950), mRNA
8	NM_105138.5	Arabidopsis thaliana with no lysine (K) kinase 10 (WNK10), mRNA
14	NM_179077.2	Arabidopsis thaliana S-adenosyl-L-methionine-dependent methyltransferases superfamily protein (ERD3), mRNA
15	NM_126596.4	Arabidopsis thaliana NagB/RpiA/CoA transferase-like superfamily protein (AT2G05830), mRNA
16, 30, 37	NM_118301.7	Arabidopsis thaliana DERLIN-2.1 (DER2.1), mRNA
17	AF088281.1	Arabidopsis thaliana phytochrome-associated protein 1 (PAP1) mRNA, complete cds
20	NM_115612.3	Arabidopsis thaliana seed imbibition 2 (SIP2), mRNA
24	AY080788.1	Arabidopsis thaliana putative phosphate/phosphoenolpyruvate translocator precursor protein (At5g33320) mRNA, complete cds
27	NM_124798.4	Arabidopsis thaliana plastid transcriptionally active 15 (PTAC15), mRNA
29	NM_124404.4	Arabidopsis thaliana chloroplast RNA-binding protein 31B (CP31B), mRNA
31	NM_001345120	Arabidopsis thaliana microtubule-associated proteins 65-1 (MAP65-1), mRNA
32	NM_127704.3	Arabidopsis thaliana B-box zinc finger family protein (BBX18), mRNA
33	LR782546.1	Arabidopsis thaliana genome assembly, chromosome: 5

34	LR782543.1	Arabidopsis thaliana genome assembly, chromosome: 2
38	NM_113511.6	Arabidopsis thaliana Plastid-lipid associated protein PAP / fibrillin family protein (AT3G26070), mRNA
39	NM_202725.1	Arabidopsis thaliana RING/U-box superfamily protein (AT3G58030), mRNA
40	AK226411.1	Arabidopsis thaliana mRNA for ferredoxin-NADP+ reductase (At5g66190), complete cds, clone: RAFL06-08-B20

Table S2. Primers in the study

Primer name	Primer sequence (5` to 3`)	Primer use
35S-FAR1-eGFP-F	acgggggacgagctcggtaccATGGCCACTCTTCCGTTGAGC	pCAMBIA1300-35S:Ab-FAR-1$^{\triangle sp}$:eGFP
35S-FAR1-eGFP-	gcccttgctcaccatgtcgacGTTCTCTTGTTTGATAAGTCCTTGA	
35S-ADF3-m-F	gagaacacgggggactctagaATGGCTAATGCAGCATCAGGA	pBI121-35S:AtADF3:mcherry
35S-ADF3-m-R	aagggactgaccacccggggaGGCTCGGCTTTTGAA	
35S-FAR1-F	acgggggacgagctcggtaccATGGCCACTCTTCCGTTGAGC	pCAMBIA1300-35S:Ab-FAR-1$^{\triangle}$
35S-FAR1-R	gcatggaagatcttcgtcgacTTAGTTCTCTTGTTTGATAAGTCCTTG	
Far1-RNAi-SF	tttggagaggacacgctcgagTACGCCGAACTTACCGATGAA	pFGC5941 (Sense, RNAi)
Far1-RNAi-SR	tcgattgggcgcgccccatggCAAGTCTGGCTTCTCTTCACCC	
Far1-RNAi-AF	ggtcttaattaactctctagaTACGCCGAACTTACCGATGAA	pFGC5941(Antisense, RNAi)
Far1-RNAi-AR	aatttgcaggtatttggatccCAAGTCTGGCTTCTCTTCACCC	
PGBKT7-far1-F	atggccatggaggccgaattcGCCACTCTTCCGTTGAGCAT	pGBKT7:Ab-FAR-1$^{\triangle sp}$(Y2H)
PGBKT7-far1-R	ccgctgcaggtcgacggatccTTAGTTCTCTTGTTTGATAAGTCCTT	
pGADT7-ADF3-F	gtaccagattacgctcatatgATGGCTAATGCAGCATCAGGA	pGADT7:ATADF3(Y2H)
pGADT7-ADF3-R	atgcccacccgggtggaattcATTGGCTCGGCTTTTGAA	
FAR1-SPYNE-F	acgggggacgagctcggtaccATGGCCACTCTTCCGTTGAGC	SPYNE-35S (BiFC)
FAR1-SPYNE-R	gcatggaagatcttcgtcgacTTAGGCCATGATATAGACGTTGTGG	
ADF3-SPYCE-F	acgggggacgagctcggtaccATGGCTAATGCAGCATCAGGA	SPYCE-35S (BiFC)
ADF3-SPYCE-R	gtacatcccgggagcggtaccATTGGCTCGGCTTTTGAA	
ADF3pro-GUS-F	ttacgaattcccggggatccTAAATGAATTTTTTTACGGGAC	pCAMBIA1381Z:AtADF3pro:GUS
ADF3pro-GUS-R	agtgccaagcttggctgcagGGTTGAATCAAAGCTAGTCTC	
fABD2-F	GATCCTCTTGAAAGAGCTGAAT	fABD2(actin marker gene)
fABD2-R	CTATTCGATGGATGCTTC	
Act1-F	ATGGCTGATGGTGAAGACATTCA	PCR detection
Act1-R	TCAGAAGCACTTCCTGTGAACAA	
hph-F	TGCGCCCAAGCTGCATCAT	PCR detection
hph-R	TGAACTCACCGCGACGTCTGT	
Hph-DIG-F	GTCTGCTGCTCCATACAA	Southern-blot probe
Hph-DIG-F	AAGGAATCGGTCAATACACT	
LBb1.3	ATTTTGCCGATTTCGGAAC	PCR detection (mutant)
LP	TTTCAGCTTGCAGTCATCATG	
RP	TCAGAAGTTTGAAACAAACAGC	
ADF3-CF	GACTCCATTATTCTCCTGCTTC	PCR detection (mutant)
ADF3-CR	GCATCACTGAGTAATAGTACGA	
qPCR1-F	TCGCTCTTCTGTCTTGCCATG	RT-qPCR(*Ab-far-1*)
qPCR1-R	GATGGATTTGTCTTCATCGGTA	
qPCR2-F	TATCCCGATGCTATGGAAACG	RT-qPCR(*Ab-far-2*)
qPCR2-R	TTTCGTCTTTGGGTTTGATGT	

qPCR3-F	TGCCCGAAGACTTGCTCGATTTCT	RT-qPCR(*Ab-far-3*)
qPCR3-R	TCGCCATCATTGCCGTTCATTGC	
qPCR4-F	ACATCCCATTGGCTATTTCCC	RT-qPCR(*Ab-far-4*)
qPCR4-R	CATTCGATCCGTGCTTCTTGA	
qPCR5-F	CACCTTGCTGGAATTGCCTGAAGA	RT-qPCR(*Ab-far-5*)
qPCR5-R	CCACCACACTTCTCAGTTGCTTCC	
qPCR6-F	GCAACAGAAGTTTGTGGCTCAACG	RT-qPCR(*Ab-far-6*)
qPCR6-R	CGGCGAATCGACGGAATGTGATG	
qPCR7-F	GGAGGAAATCGTGCCTAAGGAAGT	RT-qPCR(*Ab-far-7*)
qPCR7-R	TTGCCGAGAACCTGTTGAAGAACC	
qPCR8-F	CGACAGCCAATCCGACTCAACCAA	RT-qPCR(*Ab-far-8*)
αPCR8-R	CAAGACCAGCGGGCGAGTTCAA	
Ab-18S-F	CCGAAAGGAGATGGCAAAC	RT-qPCR(*Ab-18S*)
Ab-18S-R	ACAAAGGGCAGGGACGTAA	
Ab-ubc-F	CACAACTATCCCTACGAACCAC	RT-qPCR(*Ab-ubc*)
Ab-ubc-R	AGCGAAATAACACTCAACAAGA	
AtFRK1-F	GGGTCAGATTTCAACAGTTGTC	RT-qPCR
AtFRK1-R	AATAGCAGGTTGGCCTGTAATC	
AtPHI1-F	TTGGTTTAGACGGGATGGTG	RT-qPCR
AtPHI1-R	ACTCCAGTACAAGCCGATCC	
AtWRKY33-F	GTGATATTGACATTCTTGACGA	RT-qPCR
AtWRKY33	GATGGTTGTGCACTTGTAGTA	
AtNHL10-F	TTCCTGTCCGTAACCCAAAC	RT-qPCR
AtNHL10-R	CCCTCGTAGTAGGCATGAGC	
AtCYP81F2-F	AAATGGAGAGAGCAACACAATG	RT-qPCR
AtCYP81F2-R	ATCGCCCATTCCAATGTTAC	
AtUBP22-F	GCCAAAGCTGTGGAGAAAAG	RT-qPCR
AtUBP22-R	TGTTTAGGCGGAACGGATAC	(Reference gene)
